# Endospore pili: Flexible, stiff, and sticky nanofibers

**DOI:** 10.1016/j.bpj.2023.05.024

**Published:** 2023-05-22

**Authors:** Unni Lise Jonsmoen, Dmitry Malyshev, Rasmus Öberg, Tobias Dahlberg, Marina E. Aspholm, Magnus Andersson

**Affiliations:** 1 Department of Physics, Umeå University, Umeå, Sweden; 2 Umeå Centre for Microbial Research (UCMR), Umeå, Sweden; 3 Department of Paraclinical Sciences, Faculty of Veterinary Medicine, Norwegian University of Life Sciences (NMBU), Ås, Norway

## Abstract

Species belonging to the *Bacillus cereus* group form endospores (spores) whose surface is decorated with micrometers-long and nanometers-wide endospore appendages (Enas). The Enas have recently been shown to represent a completely novel class of Gram-positive pili. They exhibit remarkable structural properties making them extremely resilient to proteolytic digestion and solubilization. However, little is known about their functional and biophysical properties. In this work, we apply optical tweezers to manipulate and assess how wild-type and Ena-depleted mutant spores immobilize on a glass surface. Furthermore, we utilize optical tweezers to extend S-Ena fibers to measure their flexibility and tensile stiffness. Finally, by oscillating single spores, we examine how the exosporium and Enas affect spores’ hydrodynamic properties. Our results show that S-Enas (*μ*m-long pili) are not as effective as L-Enas in immobilizing spores to glass surfaces but are involved in forming spore-to-spore connections, holding the spores together in a gel-like state. The measurements also show that S-Enas are flexible but tensile stiff fibers, which support structural data suggesting that the quaternary structure is composed of subunits arranged in a complex to produce a bendable fiber (helical turns can tilt against each other) with limited axial fiber extensibility. Finally, the results show that the hydrodynamic drag is 1.5 times higher for wild-type spores expressing S- and L-Enas compared with mutant spores expressing only L-Enas or “bald spores” lacking Ena, and 2 times higher compared with spores of the exosporium-deficient strain. This study unveils novel findings on the biophysics of S- and L-Enas, their role in spore aggregation, binding of spores to glass, and their mechanical behavior upon exposure to drag forces.

## Significance

Spore-forming bacteria are a burden in our society because they can cause food spoilage and some species are pathogenic to humans. Bacterial spores attach strongly to many sorts of surfaces, they can survive high temperatures, and are resistant to a range of disinfection treatments, making cleaning and disinfection challenging in hospitals and food production industries. In this work, we characterize the biophysical properties of pili that mediate adhesion of *B. cereus* spores to surfaces. We investigate their role in aggregation, reveal their force-extension response, and influence on the spores’ hydrodynamic coefficient. This force spectroscopy study of Ena pili, known to have unique chemical properties, may contribute to the development of novel strategies to combat spore-adherence-related problems in food production and medical facilities.

## Introduction

The ability to form spores is a remarkable bacterial survival strategy used by bacteria belonging to the classes *Bacilli* and *Clostridia*. The spores are metabolically dormant, survive without nutrients and water for up to thousands of years, and are extremely resistant to stressors such as starvation, antimicrobials, wet heat, UV and gamma radiation, strong acids and bases, as well as other harsh chemical conditions. When growth conditions improve, the spores revive and result in vegetative cells capable of growing and multiplying (1,2). The *Bacillus cereus* sensu lato group (*B. cereus*) consists of several species whose spores are problematic in the food industry. Due to their wide presence in the environment, particularly in soil, *B. cereus* spores are frequent contaminants of raw food material and food production equipment. Their presence in food production chains results in large economic losses to the food producers due to: 1) the need for extensive disinfection procedures to remove spores from production equipment and tubing, 2) reduced product quality leading to reduced shelf life and food waste, and 3) resources spent on product quality and food safety measurements (3,4). *B. cereus* spores can adhere to almost all types of surfaces in food processing plants such as stainless steel, Teflon, and glass (5,6,7,8) and the spores are much more adhesive compared with their vegetative counterparts. When surface-attached spores revive, they may give rise to a growing population of bacteria capable of forming a biofilm, which could result in persistent product contamination (9,10). *B. cereus* have also been identified as a problematic pathogen within clinical environments, causing multiple outbreaks within hospitals due to their high adhesiveness to surfaces and ability to withstand decontamination (11,12). The spores’ excellent adhesion properties result from spores’ physicochemical and biophysical properties that relate to their hydrophobicity, surface charge, and surface structure (13,14,15). The biophysicochemical properties of *B. cereus* spores have been thoroughly investigated and, although there is some variation between strains, *B. cereus* spores are generally hydrophobic in nature (zeta potential of 10–40 mV [surface charge]) (7,16,17,18).

Spores’ biophysicochemical properties and extreme robustness toward environmental stressors are functions of their composition. They consist of an inner dehydrated core, containing DNA and other components needed to initiate bacterial growth when the dormancy is broken. The core is encased by several sublayers with different protective functions (19). In *B. cereus* spp. the spores are enclosed by an outermost loose-fitting paracrystalline layer, called the exosporium (20). The exosporium mediates connection of the spore to the environment and surfaces inside the infected host. In *B. cereus* spp. the spores are also decorated with micrometers-long, filamentous structures called endospore appendages (Enas) (Figs. S1 and S2). It has been suggested that the Enas facilitate adhesion of spores to both biological and abiotic surfaces, thereby promoting the first step in biofilm formation (21,22,23).

**Figure 1:**
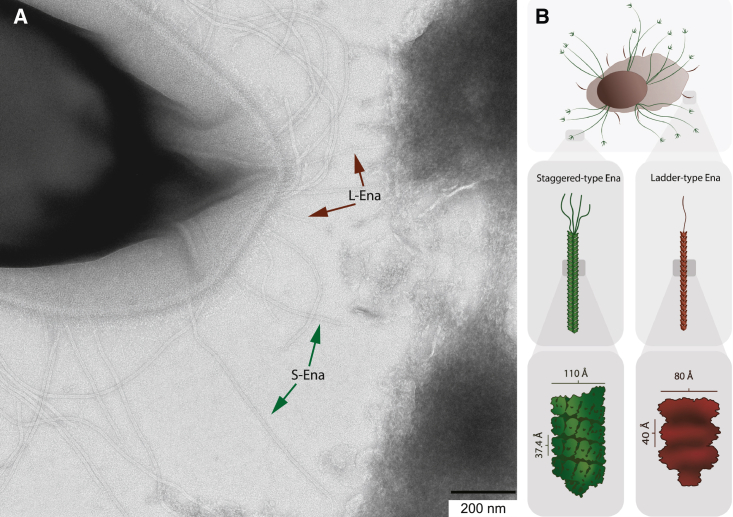
(*A*) Electron microscopy micrograph of a bacterium expressing S-Ena pili (*green arrows*) and L-Ena pili (*red arrows*). Scale bar, 200 nm. (*B*) A schematic showing the layout of a spore. To see this figure in color, go online.

Despite being observed on spore surfaces of *B. cereus* spp. since the 1970s, the structure, composition, and function of the Enas have remained unknown until recently (24), largely due to their extreme robustness toward proteolytic digestion and solubilization, which made characterization with traditional methods such as N-terminal sequencing infeasible using current techniques. However, the use of cryoelectron microscopy for structural determination forced a recent breakthrough in the structural determination of these fibers. Two morphologically different types of Enas were identified on the surface of the food poisoning outbreak strain *B. paranthracis* NVH 0075/95 (formerly *B. cereus*). Based on their appearance, they were denoted ladder (L)-type and staggered (S)-type Enas (Fig. 1 *A*). The L-Enas (*red arrows*) are hundreds of nm long, whereas S-Enas (*green arrows*) are thicker and several micrometers long. The S- and L-Ena fibers are composed of protein subunits, and both carry tip fibrilla at the end distal to the spore body (24). The protein composition and genetic identity of the tip fibrilla (adhesin) are yet to be determined (Fig. 1 *B*).

S-Ena fibers belong to a novel pilus superfamily built of two structurally similar protein monomers, denoted Ena1A and Ena1B, organized into a helical shape that is stabilized longitudinally by intermolecular disulfide bridges. Structural analysis of the S-Ena also showed a fiber with axial flexibility. The architecture provides S-Ena fibers with unique physicochemical properties including an extraordinary ability to withstand boiling, autoclaving, desiccation, proteases, and strong reducing agents such as 8 M urea or 6 M guanidinium chloride (24).

In comparison with the S-Ena, CFA/I is a pilus type commonly expressed by vegetative bacteria causing food poisoning. CFA/I pili are, for example, important for binding of enterotoxigenic *E. coli* to host intestinal epithelial cells. The protein subunits of CFA/I pili are assembled into helical-shaped rods that are 9 nm wide and a few micrometers long, i.e., similar in width and length to S-Enas (25,26). When CFA/I pili are exposed to tensile force they uncoil their helical structure and stretch up to five times their original length (27). After the tensile forces are removed, the pili regain their original shape. The superelastic property of the CFA/I pili is suggested to facilitate both initial colonization and persistent attachment to host cells. Physical models have indicated that the ability of pili to significantly extend under constant force reduces the load on the adhesin (28,29). Studies of *E. coli* suggest that pilus-mediated adherence to host surfaces is an interesting target for development of antibiotic alternatives (30,31,32). Indeed, pathogenic *E. coli* expressing mutated type 1 adhesion pili, with compromised biophysical properties, could not colonize host cells (33). Hypothetically, compromising Ena’s biophysical properties could be a strategic approach to battle *B. cereus* spp*.* spore attachment to surfaces in food production facilities. One tactic would be to develop treatments that make pili stiff or cluster together to reduce their capability to form strong interactions with these surfaces (34). However, to develop such strategies for *B. cereus*, more knowledge of S-Ena’s biophysical properties is needed.

In this work, we used force measuring optical tweezers (OT) and video analysis to characterize S-Ena’s biophysical properties and their role in adhesion. We explored the self-aggregation of wild-type (WT) and Ena-depleted mutant spores to elucidate the influence of Enas on the aggregate structure. Furthermore, we assessed the force response for single S-Ena fibers to better understand their biomechanics. Finally, we measured the hydrodynamic coefficient of *B. cereus* spores to understand how the presence of Enas affects the attachment of spores to a solid surface under a fluid flow. This study represents the first study on the function and biophysiochemical properties of genetically and structurally defined Enas. Altogether, this work provides novel insight into biomechanical properties of Enas and their role as bacterial attachment fibers. This knowledge may contribute to development of novel strategies to combat spore-adherence-related problems in food production and medical facilities.

## Materials and methods

### Strains, sample preparation, and force measurements

For our experiments we used the *B. paranthracis* NVH 0075/95 strain and five mutant strains the WT (S+ L+) strain expressing both S- and L-Enas, Δ the *ena3* (S+ L–) strain expressing only S-Enas, the Δ *ena1ABC* (S– L+) strain expressing only L-Enas, the Δ *ena1ABC* Δ *ena3* (S– L–) strain expressing neither S- nor L-Enas, the (S++ L+) strain expressing more and longer S-Enas from a low copy complementation plasmid, and finally the Δ *exsY* (exp–) exosporium-deficient strain. This strain was formerly classified as *B. cereus*, but was recently reclassified to *B. paranthracis* based on PacBio long-read sequencing and Uniclycler hydrbid assembly (GenBank: GCA_027945115.1). Spores were prepared on blood agar plates stored at 37°C for approximately 3 weeks or until the sporulation rate was 95%. We harvested the spores by scraping them off the agar surface and suspending them in autoclaved distilled water. The spore suspension was washed three times in distilled water (centrifugation at 4500 rpm for 5 min). The pellet was resuspended in 20% nycodenz (Axis-Shield) and added to a 50% and 45% (w/v) Nycodenz gradient (1:1 [v/v] ratio) before being subjected to centrifugation at 4500 rpm for 45 min. The pelleted spores were then washed once, and stored in autoclaved distilled water at 4°C until use. Before use, the concentration of the spore suspensions was adjusted to $10^{6}$ spores per mL, followed by vortexing at 2800 rpm (VM3 Vortex, M. Zipperer) for 15 s.

To attach and extend the Ena, we used surfactant-free 2.0 *μ*m aldehyde/sulfate latex microspheres (cat. no. A37299, Invitrogen, Eugene, OR) suspended in Milli-Q water. To mount the spores and reduce the influence of surface interaction, we prepared a suspension of 10 *μ*m CML latex beads (cat. no. C37259, Invitrogen) in Milli-Q water. We immobilized the 10 *μ*m microspheres onto a 24 × 60 mm glass coverslip (no. 1, Paul Marienfeld, Lauda-Königshofen, Germany) by adding 10 *μ*L of the microsphere solution onto the coverslip and drying it at 60°C for 60 min. To allow bacteria to adhere to the microspheres, we added 20 *μ*L of 0.01% poly-L-lysine solution (cat. no. P4832, Sigma-Aldrich, St. Louis, MO) to the coverslips and let them dry at 37°C for 45 min.

**Figure 2:**
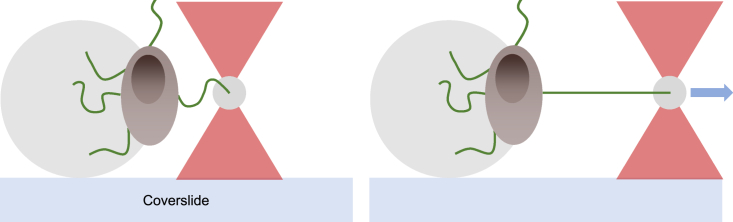
A schematic illustration of a force-extension experiment on an S-Ena pilus using OT. First, a spore is trapped with the OT and mounted on the big poly-L-lysine-coated bead. Subsequently, a microsphere is trapped and moved in proximity to the spore’s pili. After attaching the microsphere to a spore pilus, the microsphere and the spore are separated using the OT, resulting in a tensile force on the pilus. To see this figure in color, go online.

We prepared a sample by placing two strips of double-sided adhesive tape (product no. 34-8509-3289-7, 3M) close to the microsphere-coated area of our coverslips. One microliter of suspended spores was then dropped onto the area and sealed by placing a 20 × 20 mm coverslip (no. 1, Paul Marienfeld) on top of the tape. The sample was then filled completely with 10 *μ*L suspended probe beads through an open side of the sample chamber. Finally, we sealed the open sides of the sample using vacuum grease (Dow Corning, Midland, MI). The sample was then placed in an OT system. To measure the force response on a spore, we deposited the spore onto a poly-L-lysine-coated bead, attached a microsphere to the Ena, and extended the Ena using our OT system as shown in Fig. 2. To minimize the risk of attaching two or more pili to the microsphere, we probed for the longest expressed pili by positioning our trapped microsphere far away from a spore, about 3 *μ*m, and slowly moved it closer to the spore. The high force sensitivity of the OT system gives a transient signal in the force data signal when a pilus attaches to the surface. With this approach, we could, in general, adhere to the longest expressed pilus. The OT measurement procedure is described in detail in (35,36).

### OT system

To measure the biophysical properties of *B. cereus* spores, we used an OT setup built around an inverted microscope (Olympus IX71, Olympus Corporation, Japan) equipped with a water immersion objective (UPlanSApo60XWIR 60× N.A. = 1.2; Olympus) (37). We used a 1920 × 1440 pixel complementary metal oxide semiconductor camera (C11440-10C, Hamamatsu Photonics, Japan) to record samples in bright-field mode. To allow for high accuracy and precision measurements, the OT setup is optimized to minimize noise and drifts using the Allan variance method as described in (38) and the setup is positioned in a temperature-controlled and isolated room. For trapping, we use a 1064 nm DPSS laser (Rumba, 05-01 Series, Cobolt AB, Sweden). When trapping spores we made sure that the irradiation dose was below the threshold for disrupting the spore bodies, about 50 J (39).

Scattered light by the trapped object is imaged onto a 2D position-sensitive detector (2L10YAG SU65 SPC02, Sitek Electro Optics, Sweden). The centroid of the interference pattern is imaged by the position-sensitive detector and converted to a voltage signal, which is filtered with a programmable low-pass anti-aliasing filter (LTC1064-2, Analog Devices, Wilmington, MA) before measuring it using a computer equipped with a data acquisition card. We then process the collected data using an in-house-written LabVIEW program that is available upon request.

Calibration of the spring constant of the OT was done using the active power spectrum method in which a trapped microspheres’ position was sampled at 131,072 Hz for 0.25 s while being oscillated at 32 Hz. An average of 32 consecutive data sets was used for the final calibration (40). To extend an Ena fiber, we translate the piezo stage (P-561.3CD stage, Physik Instrumente GmbH, Germany) at a constant speed of 100 nm/s. We sampled the position and force at 100 Hz. The temperature of the sample chamber was measured using a thermocouple, yielding a value of 24.0 ± 0.1°C. In addition, it was assumed that the viscosity of the suspension was solely dependent on temperature. Therefore, a value of 0.932 ± 0.002 mPa s was assigned to the viscosity based on the measured temperature.

To measure the drag force of single spores, a spore was trapped in water using OT and the sample chamber oscillated (32 Hz) similar to a regular bead calibration. Thus, spores were subjected to a sinusoidal fluid drag force. The hydrodynamic coefficient for each trapped spore could then be derived by measuring the spore displacement in the trap as described in (41).

### Cell counting and immobilization assay

To determine the number of spores that were stuck to glass surfaces, we created a sample by diluting spores at a ratio of 1:10 in water and added a 10 *μ*L droplet of the solution onto a coverslide. Then, we sealed the droplet with a grease ring and a top coverslide and left it to incubate for precisely 5 min. We then recorded video sequences for three different areas of the sample, each lasting for 10 s. We analyzed in total ∼5500 spores, ∼600–1300 spores for each strain, in two biological replicate studies.

Next, we analyzed the videos to count the spores that were immobilized and those that were moving. To count the spores in each video frame, we used ImageJ software. We loaded the video frame into ImageJ, converted it into a 16-bit grayscale image (Fig. S3 *A*), and used the Otsu thresholding algorithm to separate the spores from the background. We then converted the image into a binary mask, where spores appeared white and the background appeared black. However, some spores were dark in the center, so we used the fill holes algorithm to complete these spores. From the mask, we used the analyze particles algorithm in ImageJ to count the spores by setting the size (pixel^2^) between 250 and infinity, and the circularity to 0.10–1.00. This provided the final counting mask (Fig. S3, *B* and *D*).

To count the moving spores, we used a background subtraction algorithm on the videos (42), which removed all the immobilized spores and highlighted the moving ones (Fig. S3 *C*). We then created another counting mask (Fig. S3 *D*) from the resulting image and counted the number of moving spores. Thus, by subtracting the number of moving spores from the total number we could derive the percentage of immobilized spores.

### Modeling the S-Enas using an extensible worm-like chain

Force-extension curves of S-Ena pili were described by the extensible worm-like chain (eWLC) model (43). This model takes into account both the entropic and enthalpic contributions of a polymer and the force-extension relationship can then be described by the model function,

(1) $$x=L_{0}\left(1-\frac{1}{2}\left(\frac{kT}{FP}\right)+\frac{F}{S}\right)\text{,}$$

in which *x* is the extension of the polymer, $L_{0}$ is the contour length, *kT* is the thermal energy, *F* the force, *P* the persistence length, and *S* the stretch modulus. We fitted Eq. 1 using a nonlinear optimization algorithm written in MATLAB (MATLAB R2022, The MathWorks, Natick, MA) to the force-extension data. To account for possible offsets in the force data we added an offset parameter to the fitting procedure and, to minimize any nonlinear effects of the OT force response, we limited the fitting procedure to forces up to 50 pN. To assess the length of the pili during force measurements, we measured the distance between the edge of the trapped microsphere and the edge of the spore in calibrated micrographs. Furthermore, we calculated the spring constant of a pilus by fitting a linear model to the linear portion of the force-extension data, similar to previous research (44). All parameters estimated from force-extension data are reported as mean ± SE.

The statistical analysis of the measured parameter values and the violin plots were done using GraphPad Prism 9 (Prism 9.3, GraphPad Software).

### Electron microscopy imaging

To perform SEM imaging, we air-dried a drop of spore suspension on a glass slide. The sample was then coated with a ∼5 nm layer of platinum using a Quorum Q150T-ES sputter coater. We imaged the samples using a Carl Zeiss Merlin FESEM electron microscope, utilizing the InLens and SE-2 imaging modes at a magnification of 20,000×.

For TEM we placed a copper grid (400 mesh) covered with FCF400-CU Formvar Carbon Film on top of a spore suspension droplet for 1 min. We then removed the excess suspension by capillary force using a dry filter paper. The grid was transferred to a droplet of 4% uranyl acetate for negative staining for 1 min. Once the grid had aired dried it was ready for TEM imaging using a JEM-2100Plus Electron Microscope (JEOL Ltd., Japan).

### Construction of the exosporium-depleted mutant strain

The *exsY*-depleted mutant was constructed as described by Pradhan et al. (24) using markerless gene replacement (45) in the *B. paranthracis* NVH 0075/95 background strain. We used PCR to amplify the flanking fragments of *exsY* using chromosomal DNA as a template and the primer pairs A/B and C/D (Table S1). The PCR products were then fused together by PCR using the A and D primers. The resulting fragment was ligated into a pMAD vector and cloned into One Shot TOP10 chemically competent *E. coli* (Invitrogen, Thermo Fisher Scientific, USA) for plasmid amplification. The construct was verified by sequencing (LightRun, Eurofins, Luxembourg). We passed the final construct through One Shot INV110 *E. coli* (Thermo Fisher Scientific) for demethylation of the vector before it was transformed into electrocompetent cells of *NVH 0075/95*. Colonies containing the correct construct, i.e., a chromosomally integrated pMAD construct, as verified by PCR, were transformed with a demethylated pBKJ223 plasmid. The pBKJ223 plasmid encodes an enzyme that introduces a double-stranded DNA break into the chromosomally integrated pMAD plasmid to stimulate its excitation from the chromosome, leaving a markerless target gene deletion behind (24). The correct modification was verified by PCR of regions upstream and downstream of the gene of interest using chromosomal DNA from the transformant clones as a template and the E/F primer combination. The exosporium-deficient phenotype was confirmed by TEM imaging of the mutant spores as seen in Fig. S4.

## Results and discussion

### Enas role in glass and spore-to-spore attachment

To evaluate the role of Enas in attachment to glass and spore aggregation, we assessed how spores were immobilized on glass coverslides and how spores formed interactions and aggregates in water. Moreover, we employed the OT technique to manipulate spores and quantitatively assess their pili force response, thus gaining insight into their intrinsic biophysical properties. The following strains were included in the analysis: the *B. paranthracis* NVH 0075/95 WT strain expressing both S-Enas and L-Enas (S+ L+) and its isogenic Δ *ena3* mutant only expressing S-Enas (S+ L–), the Δ *ena1ABC* mutant expressing only L-Enas (S– L+), the Δ *ena1ABC* Δ *ena3* mutant expressing neither S-Enas nor L-Enas (S– L–), the complemented strain (S++ L+) expressing longer S-Enas, and finally the Δ *exsY* exosporium-deficient mutant (exp–).

**Figure 3:**
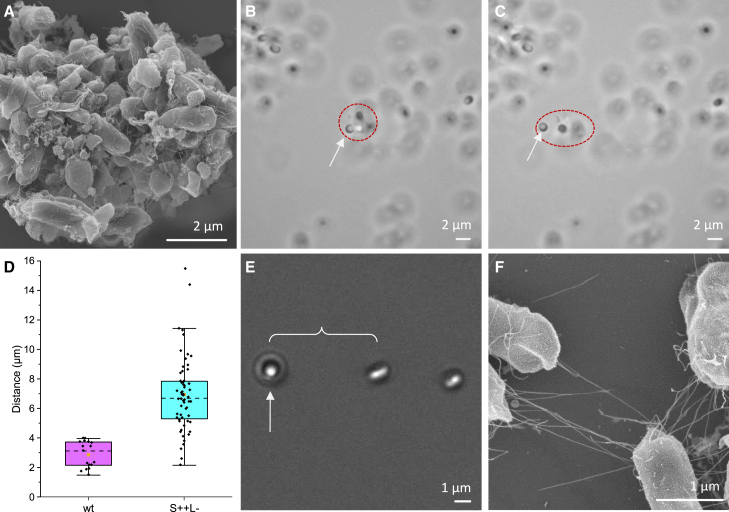
(*A*) SEM image of aggregated WT (S+ L+) spores. (*B*) A single trapped WT spore (*white arrow*) attached to a spore aggregate (*dashed red circle*). (*C*) We displaced the trapped spore to the left and measured the distance between the spores as well as the force needed to move it. (*D*) The boxplots show the first and third quartiles (*box limits*), mean values (*orange squares*), median values (*dashed lines*), and 1.5 interquartile range (IQR) (whiskers) for the distance between interconnected WT spores. (*E*) Bright-field images showing the (S++ L+) mutant with extra long S-Enas forming a “string of pearls” when pulling on one spore (*white arrow*) that is connected to others. (*F*) SEM image showing multiple interconnected pili between spore bodies. To see this figure in color, go online.

To investigate the behavior of WT and mutant spores on glass surfaces, we conducted two experiments. First, we added the spores to a coverslide and used video recording to observe their Brownian motion. Second, we used OT to manipulate the spores to see if they could be trapped and moved. The results showed that spores lacking the exosporium (exp–) form tight aggregates and exhibit spore-to-spore adhesion as well as strong binding to glass coverslides (21% were immobilized). Immobilized spores could not be removed from the coverslides using the OT operating with a high trap stiffness of around 1000 pN/*μ*m (see Video S1). Moving a trapped spore close to other spores did not indicate that they formed distant connections. Spores with an intact exosporium but lacking both S- and L-Ena (S– L–) also attached to the glass coverslide (43% immobilized), but could, unlike their exosporium-deficient counterparts, be trapped and moved from the glass using the OT. We observed that many of the (S– L–) spores were floating on top of spores residing on the glass surface and could also be trapped or blasted away by the radiation pressure (see Video S2). These spores did not clump together as the (exp–) and did not form long-range spore-to-spore connections. This suggests that the exosporium plays a role in spore spread by inhibiting the spores from forming excessively compact aggregates. Spores expressing only L-Ena (S– L+) were rarely found as free-floaters but, instead, a majority were found to be immobilized (66%) to the glass slide (see Video S3). They could not be removed by the OT, indicating that the binding was strong. Also, spores did not form long-range connections and were limited in aggregation. The WT (S+ L+) spores that had settled on the glass slide showed a high degree of immobilized spores (71%); however, they could be moved (not removed) by the OT, indicating that they were tethered but not immobilized as the (S– L+). They also formed less compact aggregates, resembling a gel-like structure in which spores were more distant apart but could still attach to each other (see Fig. 3 *A* and Video S4). The (S+ L–) mutant also formed less compact aggregates, and fewer spores were immobilized (35%) to the glass. We also noticed that when trapped spores were moved by the OT in proximity to other spores, they attached to each other and formed a train resembling a “pearl string” (Video S5). Such a pearl string appearance was seen also for the (S++ L+) mutant that formed extra long S-Enas (Video S6). However, these spores were positioned more distant apart than for the (S+ L–). In general, the (S++ L+) mutant spores were not tightly packed, and only 21% were immobilized, indicating that these spores are more prone to “float” on the surface, most likely due to the long-expressed pili displacing the spores’ bodies from the surface preventing L-Enas to firmly attach them.

Video S1. Strain lacking exosporium (exp–, lab. strain name 1521)

Video S2. Strain lacking S- and L-Enas (S– L–, lab. strain name 1500)

Video S3. Strain lacking S- but expressing L-Enas (S– L+, lab. strain name 1489)

Video S4. Strain expressing S- and L-Enas (S+ L+, lab. strain name 160)

Video S5. Strain lacking L- but expressing S-Enas (S+ L–, lab. strain name 1503)

It is not known whether the connection between spores in the gel-like structure of the aggregates is mediated via the exosporium or via Ena pili. We hypothesized that if the exosporium or L-Ena pili mediate the binding, the center-to-center distance between spores should be at most 1–2 *μ*m since the exosporium reach about 500 nm (see Fig. S5) and the lengths of L-Enas are about 200 nm (see Fig. 1). Therefore, to investigate the physical connection between the spores, we trapped single spores within the gel-like aggregate and measured the distance as well as the applied force needed to move them using OT. An example of a trapped WT spore (*dashed red circle*) that is moved about 10 *μ*m to the left (*dashed red circle*) is seen in Fig. 3, *B* and *C*. We observed that, when moving the spore, a larger aggregate of spores followed. To quantify the distance between interconnected WT spores forming a pearl string, we recorded videos and measured the distance in video frames. For WT spores the average center-to-center distance was 2.9 ± 0.9 *μ*m (*n* = 17, mean ± 1 standard deviation [SD]), see Fig. 3 *D*. These results thereby indicated that spore-to-spore connections likely are mediated via the longer S-Ena pili. Moreover, to strengthen the hypothesis that binding is indeed mediated via S-Ena pili, we used a mutant expressing extra long S-Enas (S++ L+) and one that lacked the L-Enas (S+ L–). Similarly, as before, we trapped (S++ L+) spores and measured the distance between interconnected spores, indicated by the curly brackets in Fig. 3 *E*. The center-to-center distance between these mutant spores was significantly longer (*p* < 0.0001), at 7.0± 2.5 *μ*m (*n* = 60) on average, with some distances >10 *μ*m (Video S7). Finally, since the (S+ L–) formed a train resembling a pearl string (Video S5), never observed for the (S– L+) and (exp–), we could rule out that both the L-Enas and exosporium have a major role in connecting spores. The S-Ena role in the pearl string appearance was further confirmed by TEM micrographs (see Fig. S6) showing the extra long S-Enas of multiple (S++ L+) spores tangled together. Thus, these results strongly indicated that S-Enas are important for spore-to-spore connection.

Video S6. Strain expressing extra long S- and L-Enas (S++ L+, lab. strain name 1487)

Video S7. Strain expressing extra long S- and L-Enas (S++ L+, lab. strain name 1487)

**Figure 4:**
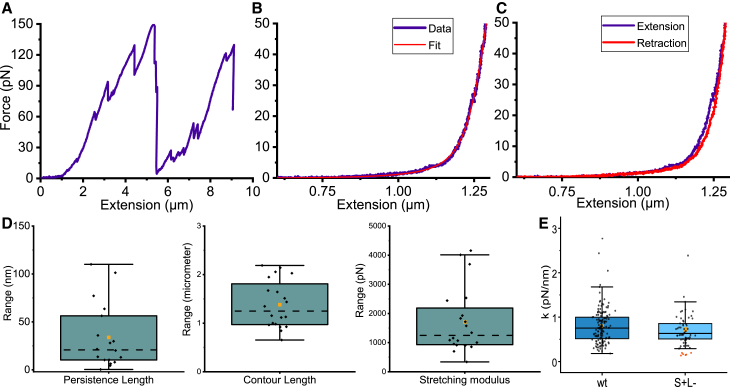
OT force measurements of WT (S+ L+). (*A*) When moving the trapped spore in Fig. 3, *B* and *C*, we can see an increasing force with extension in which several rapid force drops form a saw-tooth pattern. The saw-tooth pattern indicates the release of attachment points in the spore aggregate. (*B*) A force-extension curve (*purple*) fitted with the eWLC model (*red*). (*C*) Force extension (*purple*) and retraction (*red*) curves overlap with no significant hysteresis. (*D*) Boxplots of parameter values from the worm-like chain fit. The boxplots are defined as in Fig. 3*D* with mean values, median values, and 1.5 IQR (whiskers) for the persistence length, contour length, and stretching modulus (*n* = 18). (*E*) The boxplots show the spring constants of the WT (left) and (S+ L–) mutant (*n* = 149 and 57). Outliers from a single pilus measurement with substantial cross talk on the detector are denoted by the red dots. To see this figure in color, go online.

During the separation of spores from aggregates we also monitored the force response. An example of such force response, of the spore moved in Fig. 3 *B*, is seen in Fig. 4 *A*. Initially, the force is constant with extension and thereafter increases more or less linearly with extension but shows both small and large force drops, in the order 10 and 100 pN, respectively (for example, at 2.2, 3.0, 4.0, 5.5 *μ*m). These linear increments in force versus extension indicate that the tethers connecting spores are elastic, that is, following a Hookean spring response. Thus, these pili behave as springs when a spore is exposed to force. In addition, the force drops indicate that multiple connections were formed either spore-to-spore or pili-to-pili as seen in Fig. 3 *E*, and that these detached during pulling. It should be noted that the calibration procedure used to estimate these forces work best for spherical particles. Thus, as the spore are only approximately spherical, the absolute forces measured are not exact. However, the force data still give a good suggestion about the relative force differences between spore strains and give an approximate image of the forces needed to disconnect these spores from each other. How spores connect to each other is not known, but SEM image analysis suggests that S-Enas can bind to the exosporium surface (Fig. 3 *E*). We most likely observe S-Ena detaching from other spores; however, to pinpoint the exact mechanisms behind potential adhesin-epitope interactions we are dependent on mutants depleted of the S-Ena tip fibrilla. Due to their unknown identity, these mutants are still unavailable. Therefore, next we focused on characterizing the mechanical properties of the S-Ena shaft.

### S-Ena pili are flexible structures with high axial stiffness

The experiments above suggest that the short L-Enas play a role in enhancing adhesion to glass surfaces compared with S-Enas. On the other hand, the longer S-Enas contribute to spore-to-spore connections and spore clustering. With S-Ena present spores are also more spread apart.

Previous observation of S-Ena-expressing *B. cereus* spores indicate that they also bind well to abiotic surfaces, for example, plastic (10,46). This characteristic is clearly demonstrated when mixing spores using plastic tubes and pipette tips, *B. cereus* spores easily form thin films. Based on the strong binding observed with plastic, we decided to use polystyrene microspheres as targets for measuring the binding of S-Enas in our OT pili assay. We applied a tensile force of up to 150 pN to a pilus fiber and found that S-Ena fiber displayed strong adhesion to the microspheres without releasing its binding. Our results showed that the interaction between S-Ena and polystyrene beads was significantly stronger than the corresponding interaction observed for the helix-like pili expressed by uropathogenic and enterotoxigenic *E. coli* (27,47). However, the interaction was similar to that observed for archaic chaperone-usher pili, which are also known to bind well to plastic surfaces (48).

To assess the dynamics, entropic elasticity, and the spring constant of S-Ena pili, we measured their biomechanical properties using OT force-extension/contraction assays. We attached a 2.0 *μ*m trapped polystyrene microsphere to a pilus, and subsequently separated the bacterium-microsphere complex by translating a piezo-stage at 100 nm/s. This applies a tensile force to the pilus under investigation, to which the force response can be assessed with sub-pN force resolution. In general, we applied up to 50 pN of force to run the laser at lower power and to avoid a nonlinear force response. An example of such an experiment is shown in Fig. 4 *B*, which shows the extension phase (*purple*) that corresponds to straightening the pilus. However, as the tension increases and the pilus is straightened close to its contour length more force is required to extend it further. A corresponding fit of the eWLC model, Eq. 1, to the force data is shown by the red solid curve. The fit provides the parameter values for the persistence length and stretch modulus, which for these particular data are 112 nm and 496 pN. The average values for all measured pili from *n* = 18 spores are 34±8 nm and 1710±250 pN (mean ± SE), data distributions are seen in Fig. 4 *D*, and additional force-extension curves with corresponding eWLC fits are shown in Fig. S7. The variability in the parameter values may stem from the force data fluctuations or interactions of two or more pili with the bead. Two measurements were identified as extreme outliers (with persistence lengths of 1346 and 452 nm), and thus were excluded from the average. Based on the measured average persistence length of 34 nm and subunit length of approximately 4 nm, it can be inferred that the structure of S-Enas does not exhibit a curled mesh arrangement on the surfaces of spores. Rather, it is likely to consist of relatively straight segments. This suggestion is in agreement with what is observed in TEM micrographs in this work (see example in Fig. 1 *A*) and in (24). The length of the eWLC curves, mean contour length of 1.40±0.11 *μ*m, also suggests that the observed force responses are from the extension of pili and not from individual pilin subunits comprising approximately 100 residues (24). These length estimations coincide reasonably well with the average length of WT S-Enas of 1.27±0.07 *μ*m (mean ± SE), gathered by measuring 46 S-Enas in TEM micrographs (Fig. S8).

In the force data, we further note that the force increases linearly after 1.25 *μ*m extension. This linear increase resembles the force response of a purely elastic spring indicating that S-Enas can elongate in the axial direction. The rapid increase of the force with extension and the measured stretch modulus of 1710 pN suggest a fiber with limited extensibility upon applied force. This is in agreement with the solved S-Ena structure, which shows a structure where helical turns are longitudinally stabilized via disulfide bonding (24). Such an arrangement provides indeed a fiber that is robust to tensile forces.

To investigate if the structure was damaged upon extension, we also recorded the retraction phase as seen in Fig. 4 *C*. The retraction curve closely followed the extension curve, indicating no fatigue in the Ena fiber when tensile force was applied. We also performed multiple force-extension measurement cycles on a fiber, which again showed no hysteresis or breakage of the fiber even though applying up to 250 pN (Fig. S9). Since we could not apply as high force as with magnetic tweezers or atomic force microscopes we could not measure the complete extensibility, i.e., the force required for the fibers to break or pilins to unfold. However, we can conclude that we did not observe any conformational changes or nonlinearity in the force response for the range of forces that we applied during the extension phase. Thus, no hysteresis is present at the experimental timescale and the pilus is neither damaged upon tensile force nor dissipates a significant amount of energy to the surrounding environment during an extension/contraction cycle.

As mentioned above and seen in Fig. 4, the force-extension signature at higher forces is a linear region following a Hookean spring. Similar Hookean behavior has been observed for pili expressed by Gram-positive bacteria such as *Pseudomonas aeruginosa*, *Streptococcus pneumoniae*, *Lactobacillus rhamnosus GG* (*LGG*) (49,50,51,52), and the surface-contact sensor type IVc pilus (T4cP) that is found in both Gram-positive and Gram-negative bacteria (44). To compare the spring behavior of our S-Enas with these other pili types, we therefore estimated the spring constant of the linear part of the force curves by fitting a linear model. Also, to confirm that the interactions we observe are specifically from the S-Enas, rather than a combination or interplay of S-Enas and L-Enas, we also performed force-extension measurements (*n* = 57 technical replicates, *n* = 13 biological replicates) on L-Ena-deficient isolate spores (S+ L–) and compared them with the WT spores (*n* = 149 technical replicates, *n* = 20 biological replicates). We found that there was no statistical difference (*p* = 0.25) in the spring constant of the WT (0.79 ± 0.39 pN/nm, mean ± SD) compared with the S+ L– isolate (0.72 ± 0.34 pN/nm), indicating that our previous force-extension experiments carried out were showing the S-Ena response (see Fig. 4 *D*). To verify the similarity between S-Enas expressed by WT and (S+ L–), we assessed TEM micrographs. The (S+ L–) spores show S-Enas similar to those expressed on the WT spores (Fig. S10), and of a comparable length distribution with an average length of 1.33±0.07 *μ*m (*n* = 82, mean ± SE), as seen in Fig. S7.

With a spring constant of 0.79 pN/nm, S-Enas are not as stiff as LGG pili (broad range reported, 4–15 pN/nm), type IV pili (reported at 1.1 pN/nm (49) and 2 pN/nm (51)), and T4cP (broad distribution 1–10 pN/nm with a mean about ∼5 pN/nm). The probable reason for the lower spring constant value observed in S-Enas is the presence of a flexible spacer region between the Ena domain and the N-terminal connector (Ntc) formed by amino acid residues 12–17. This flexible spacer generates a longitudinal gap of 4.5 Å between the S-Ena subunits, which are not in direct physical contact except through the Ntc. The significant flexibility and elasticity of the S-Ena pili are a result of the flexibility of the Ntc spacer and the absence of direct protein-protein interaction among subunits across the helical turns (24).

Due to the lack of microfluidic shear force studies of spores expressing S-Enas, and the lack of knowledge of the S-Ena adhesin, we avoid speculating on the functional role of the S-Ena biophysical properties. Instead, we believe that this study opens up new exciting research questions to solve in the future. For example, the lack of knowledge of the S-Ena adhesin, how it binds and its binding type (slip or catch bond), and whether there is a correlation between the biomechanical function of the S-Ena rod and the adhesin, similar to what has been observed for type 1 pili. Furthermore, S-Ena pili is more suitable for attachment in high shear stress conditions in pipes resulting in drag forces that are significantly higher than what is observed *in vivo*, e.g., in the intestine? These are fundamental and important research questions to solve, which require better structural knowledge, physical modeling, and microfluidic measurements. To help solve these microfluidic-related questions in the future, we did a final experiment to determine if S-Enas and the exosporium influence the hydrodynamic drag coefficient on spores exposed to fluid flow.

### S-Ena and exosporium significantly increase the hydrodynamic drag of spores

*B. cereus* spores are highly hydrophobic and can firmly adhere to inert materials such as stainless steel, glass, and plastic. When attached, the spores can revive resulting in vegetative cells capable of forming robust biofilms that protect bacteria and spores from external stressors. In the food industry, *B. cereus* biofilms can act as reservoirs of spores that can lead to persistent product contamination.

**Figure 5:**
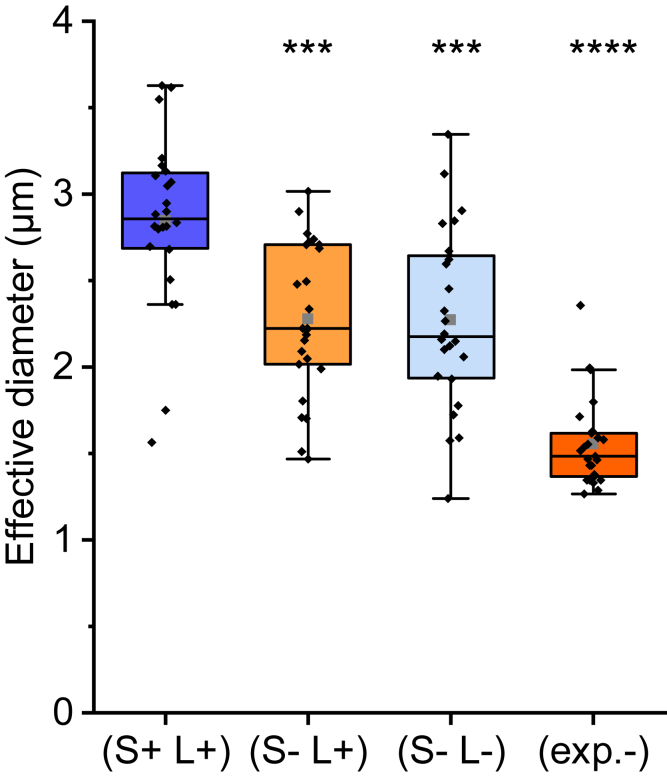
The boxplots show the effective hydrodynamic diameter of WT spore (S+ L+), spores with only L-Ena pili (S– L+), spores lacking S-Ena and L-Ena pili (S– L–) and spores lacking an exosporium (exp–). The boxplots are defines as in Figs. 3 and 4 with mean values, median values, and 1.5 IQR (whiskers).Stars indicate the statistical significance of the difference between the data sets. *n* = 25 for all data sets. To see this figure in color, go online.

Dissemination of spores under fluid flow conditions is strongly correlated with their ability to adhere to each other and to underlying surfaces (attachment strength), and to their hydrodynamic coefficient (shear stress from the fluid). To investigate if the presence of S-Enas on the spore surface affects the hydrodynamic coefficient, that is, their impact on the drag force, we measured the effective diameter of individual *B. cereus* spores using oscillating OT. This method has previously been shown to be able to quantify the degree of surface pili expressed by *E. coli* bacteria (41). To perform these measurements, we trapped single spores and oscillated the sample chamber, thus exposing spores to a sinusoidal fluid drag force. By measuring the spore displacement in the trap, we could derive the hydrodynamic coefficient for each trapped spore. The results show that spores expressing both S- and L-Enas (S+ L+) have an effective diameter of 2.84 ± 0.49 *μ*m (mean ± SD). Comparatively, spores that express no Ena (S– L–) or only L-Ena (S– L+) have a significantly lower (20%) effective diameter at 2.26 ± 0.51 and 2.27 ± 0.44 *μ*m, respectively. Spores lacking exosporium (exp–) showed an even lower (46% smaller) effective diameter at 1.55 ± 0.26 *μ*m, see Fig. 5. This is a remarkable difference, with *p* = 0.0003 for the Δ *ena1ABC* (S– L+) strain, *p* = 0.0005 for the Δ *ena1ABC* Δ *ena3* (S– L–) strain, and *p* < 0.0001 for the Δ *exsY* (exp–) strain, all compared with the WT strain (S+ L+). Together, these data show that S-Enas significantly contribute to the hydrodynamic coefficient as previously indicated in studies of spore detachment (53).

To model the role of the larger effective radius of S-Ena-expressing spores in a fluid flow we used the Goldman-corrected Stokes drag force model to simulate the drag force on spores located at various distances from a surface (see Fig. S11) (54). We found that, far from the surface (>10 *μ*m) in relation to the spore diameter ∼1 *μ*m, S-Enas increased the Stokes drag force by 27% compared with spores lacking both S- and L-Ena or spores expressing only L-Ena (S– L+). When located closer to the surface (∼1 *μ*m), this increase is 45%. From a physical point of view, we can speculate how this affects the dissemination of spores from a biofilm. The S-Ena assists in the initial formation of the biofilm by facilitating the binding of a free-flowing spore to a surface. In a more mature biofilm, consisting of layers of spores, vegetative cells, and extracellular matrix, that extends from a surface and thus experiences higher flow velocities, the increased drag force generated from the S-Ena allows individual spores in the biofilm to detach and disseminate. This method of dissemination is particularly relevant for spores, as in comparison with motile bacteria that express flagella to move in fluid, spores are passive bodies that rely on fluid flow to disseminate. Interestingly, it has been shown that *E. coli* overexpressing CFA/I adhesion pili, significantly impairs its flagella-mediated swimming capability (55). However, such an effect is not relevant for the passive spore state. Instead, we suggest that the expression of S-Ena may serve two purposes. Expression facilitates adhesion but, at the same time, increases the hydrodynamic drag coefficient making detachment and dissemination of spores from biofilms more probable.

## Conclusions

*B. cereus* spores are frequent contaminants of food raw materials and food production equipment. This is problematic to the food industry because, when spores revive, they result in vegetative cells able to cause food spoilage and food poisoning. Contamination of food production facilities and the resistance to cleaning depends largely on the great adhesiveness of *B. cereus* spores to various surfaces. Their ability to bind to abiotic surfaces is correlated with their physicochemical properties such as hydrophobicity, but our data suggest that surface appendages also promote adhesion. Recently, S- and L-Ena fibers were characterized on the surface of a *B. paranthracis* food poisoning outbreak strain. We show that these two types of Ena exhibit different binding properties, with the longer and wider S-Ena fiber accommodating binding between spores, promoting the formation of gel-like aggregates. The shorter L-Ena fibers, were, on the other hand, more important for binding spores to a glass surface compared with the S-Ena fibers. Individual S-Ena fibers exhibit a flexible structure with high axial stiffness. Force-extension measurements on S-Enas show that they exhibit a force signature well fitted by an eWLC. We further show that spores expressing S-Enas have a 30% higher hydrodynamic radius than Ena-depleted spores, making S-Ena more susceptible to hydrodynamic forces in a fluid flow. Overall, we hope this study invites more research into the previously unexplored mechanics of Enas and facilitates the development of new methods to battle persistent contamination with *B. cereus* spp. spores.

## Author contributions

T.D., M.E.A., and M.A. designed the research. T.D. and U.L.J. performed the research. T.D., D.M., R.Ö., U.L.J., and M.A. analyzed the data. M.A. and M.E.A. organized the project. All authors wrote the manuscript and have read and agreed to the published version of the manuscript.

## Supporting material

Document S1. Figures S1–S11 and Table S1

Document S2. Article plus supporting material
