# Supplementary material for: Endospore pili: Flexible, stiff, and sticky nanofibers
Source: Biophys J. 2023 May 22;122(13):2696–706. doi: 10.1016/j.bpj.2023.05.024 (PMC10397575; doi:10.1016/j.bpj.2023.05.024)
Supplement: Document S1. Figures S1–S11 and Table S1 [file mmc1.pdf]

Biophysical Journal, Volume *122*

Supplemental information

Endospore pili: Flexible, stiff, and sticky nanofibers

Unni Lise Jonsmoen, Dmitry Malyshev, Rasmus Öberg, Tobias Dahlberg, Marina E. Aspholm, and Magnus Andersson

Endospore pili - flexible, stiff and sticky nanofibers

Unni Lise Jonsmoen[c,d], Dmitry Malyshev[a,d], Rasmus Öberg[a], Tobias Dahlberg[a], Marina E. Aspholm[c, *]
Magnus Andersson[a, b, *]

[a]Dept of Physics, Umeå University, 901 87, Umeå, Sweden
[b]Umeå Centre for Microbial Research (UCMR), Umeå, Sweden
[c]Department of Paraclinical Sciences, Faculty of Veterinary Medicine, Norwegian University of Life Sciences (NMBU), P.O. Box 5003, 1432 Ås, Norway
[d]Shared first authors
*Corresponding authors: marina.aspholm@nmbu.no and magnus.andersson@umu.se

Supporting Information Contents

Supporting Information Contents 1
Additional SEM micrographs 2
Counting the number of immobilized cells 3
Genetic primers and TEM micrographs of exosporium depleted mutant spores 3
TEM micrographs of exosporium depleted B. cereus mutants (exp-) 4
TEM micrographs of wild type spores with intact exosporium 5
TEM micrographs of S++mutant spores 5
Additional force-extension curves with eWLC fits 6
Lengths of S-Ena, as measured from TEM micrographs 6
Performing multiple extension measurements 7
TEM micrographs of S+L- mutant spores 8
Drag force simulation modeling 9
Supporting Movies 10
References 10

Additional SEM micrographs

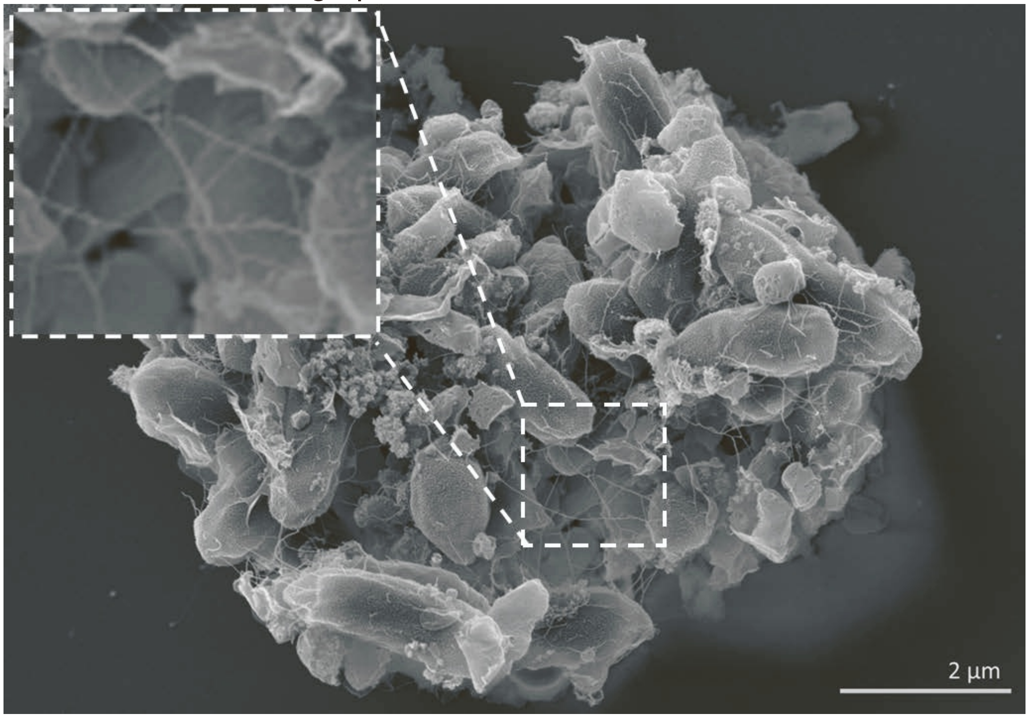

Figure S1. SEM micrograph of aggregated *B. paranthracis* wild type spores. Multiple long pili can be seen, covering and linking the spores (inset). Scale bar is 2 µm.

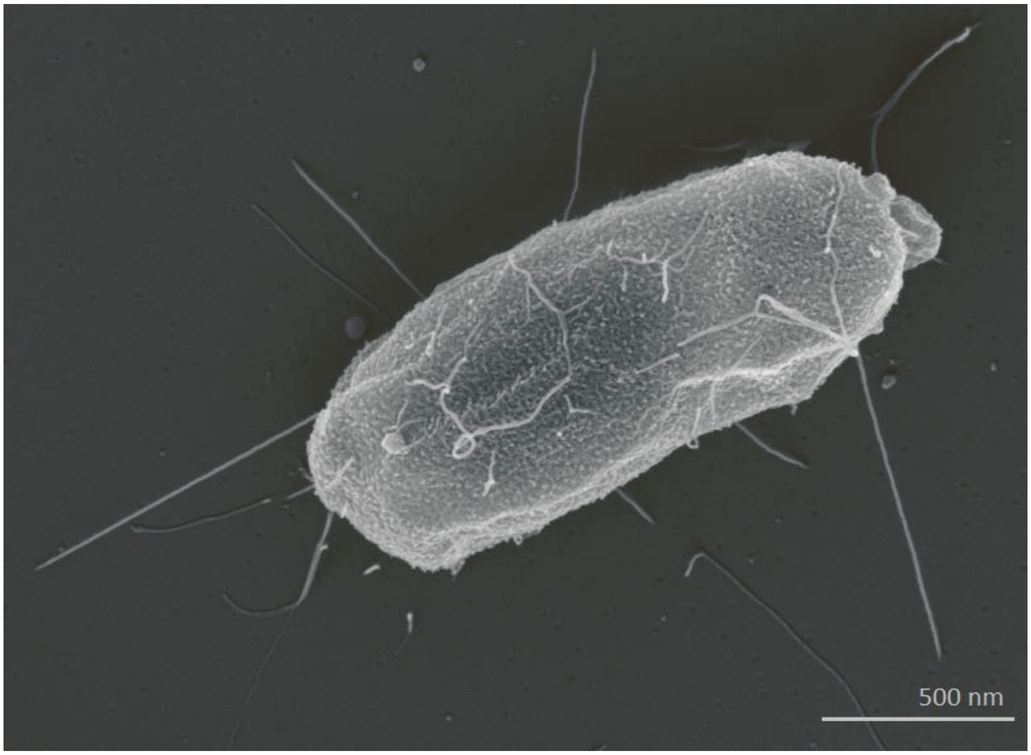

Figure S2. SEM micrograph of a single *B. paranthracis* wild type spore. Multiple stiff and relaxed pili can be seen attached to the spore. Scale bar is 500 nm.

Counting the number of immobilized cells

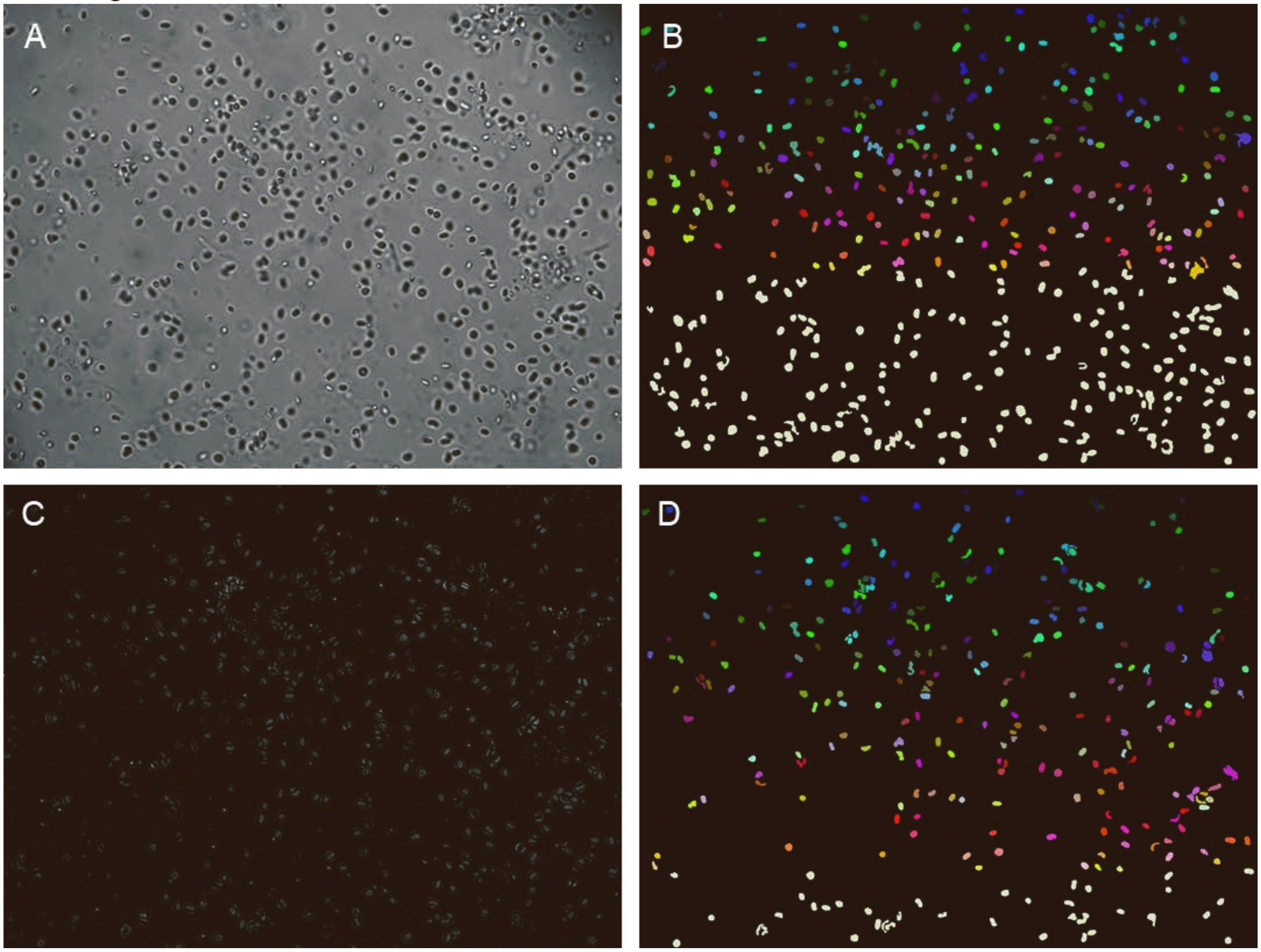

Figure S3. Counting the number of immobilized cells using ImageJ software [1]. A) shows a frame from a video sequence. B) shows the counting mask colored using 3-3-2 RGB. C) shows a frame from a background subtracted (10 second) movie indicating moving spores. D) shows the counting mask of the moving spores colored using 3-3-2 RGB.

Genetic primers and TEM micrographs of exosporium depleted mutant spores

Table S1: Primers used to construct exosporium depleted *B. paranthracis* strain.

Primer	Sequence (5'-3')
A	GCACTAACCCATACACCTGTACACC
B	CCTCACTGTAATTACATTTTTTGATAACCCCACCATCTT
C	GAGTGACATTAATGTAAAAAACTATTGGGGTGGTAGAA
D	GCAAGAGCCAGTGAGAACTGTTCCG
E	GCTCACAGCAAACATCTCCTCTACTG
F	GTAAGTAGTGCTGTGGAAGAGG

TEM micrographs of exosporium depleted B. cereus mutants (exp-)

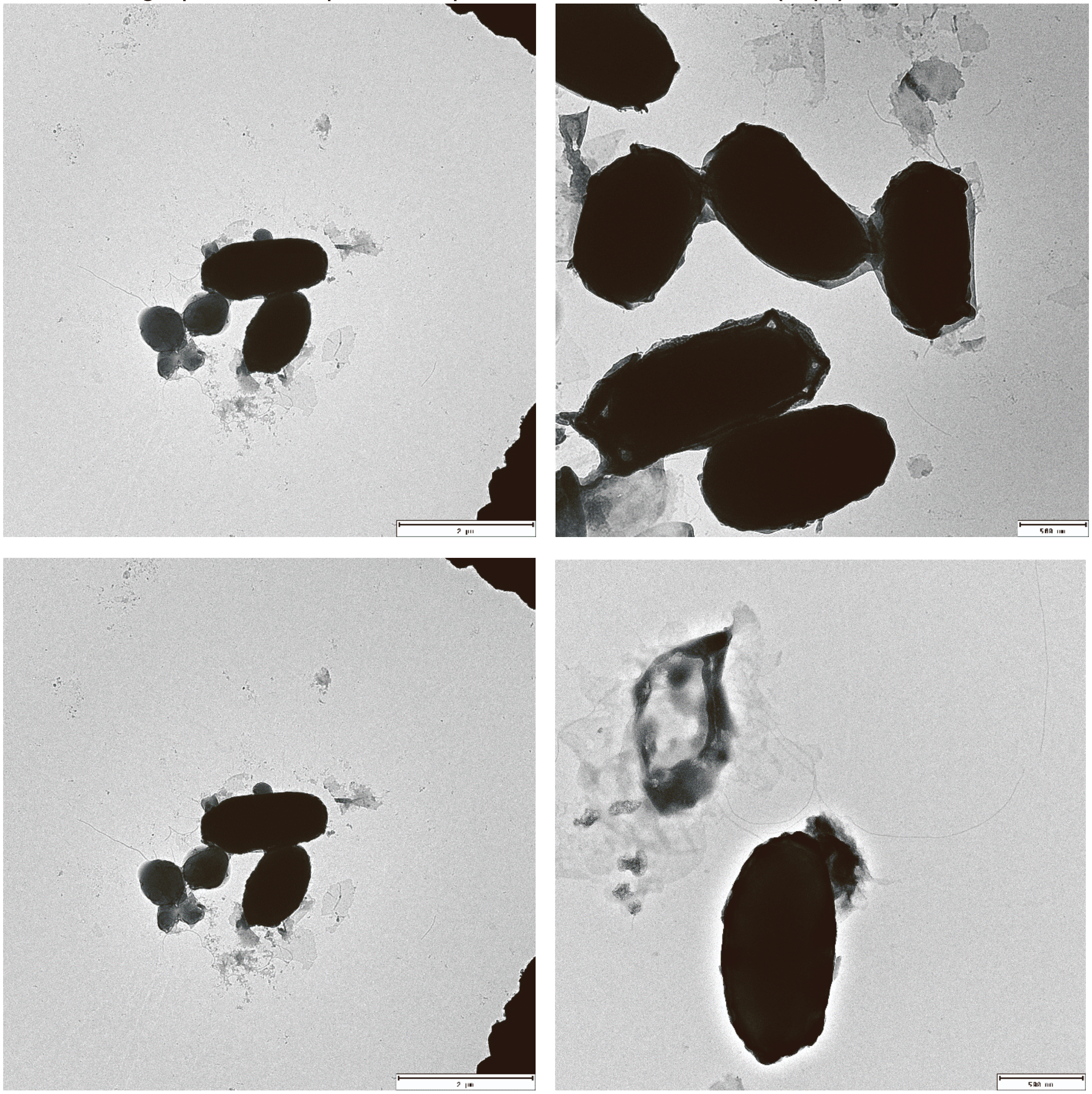

Figure S4. TEM micrographs of exosporium depleted *B. paranthracis* mutants (exp-). The spores feature no exterior layer outside the elliptical main body of the spore.

TEM micrographs of wild type spores with intact exosporium

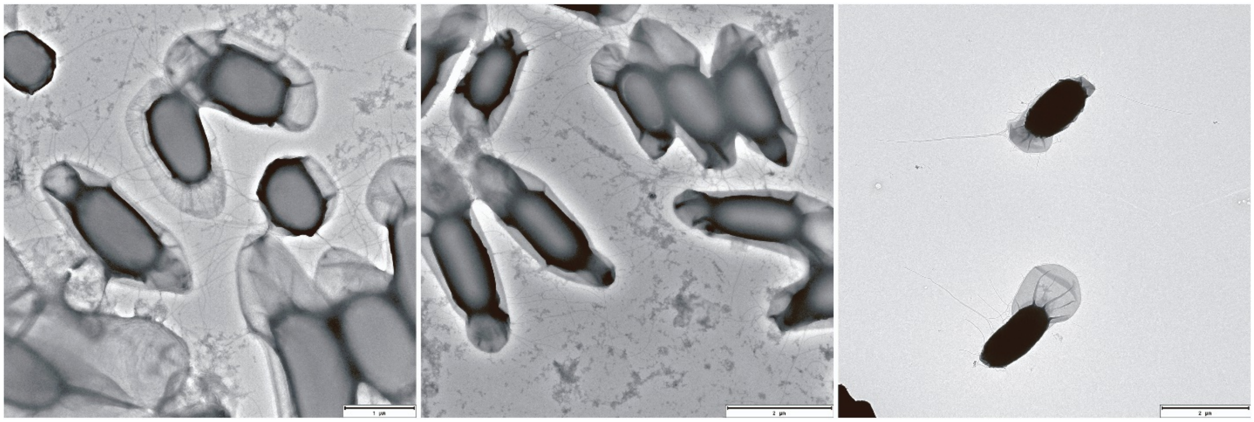

Figure S5. TEM micrographs of wild type *B. paranthracis* NVH 0075/95 spores. The distance between spore body and the distal edge of the exosporium is on average around 500 nm.

TEM micrographs of S++mutant spores

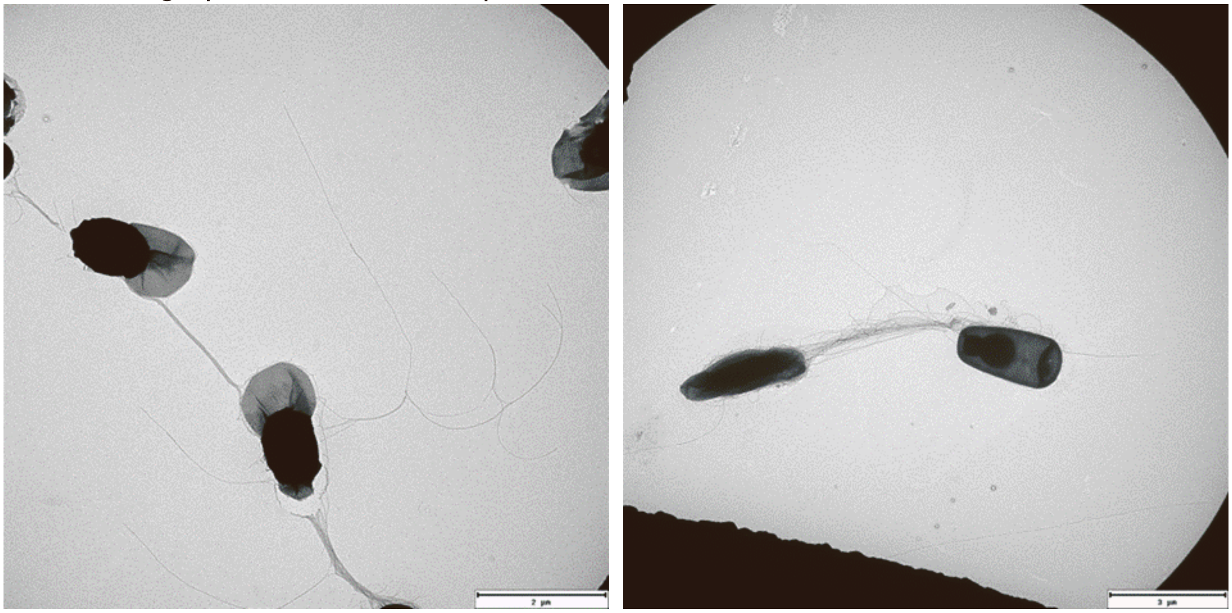

Figure S6. TEM micrographs of the S++ *B. paranthracis* NVH 0075/95 mutant strain. The spores feature more and longer S-type appendages compared to the wild type strain.

Additional force-extension curves with eWLC fits

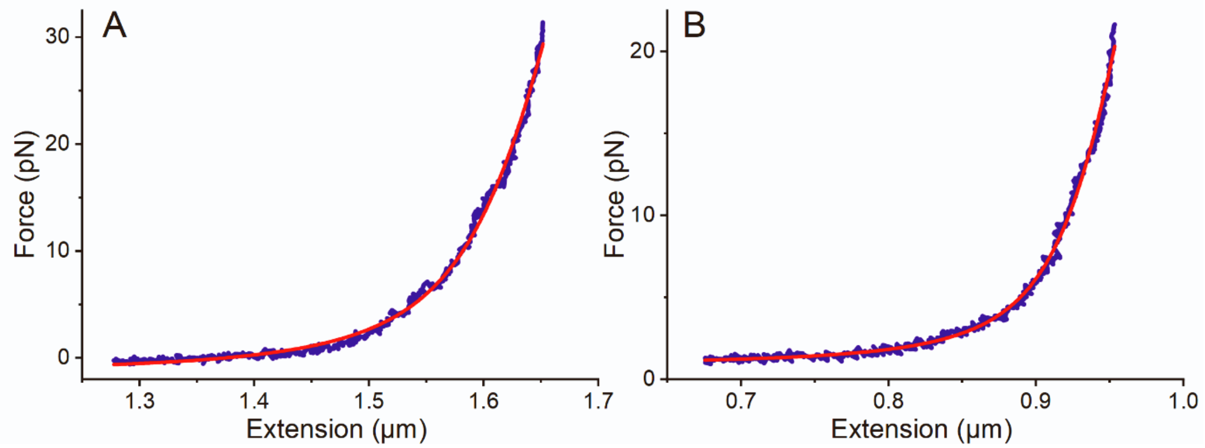

Figure S7. Additional representative force-extension curves (purple) of S-Ena on wt spores with corresponding eWLC fits (red). The fitted persistence length, contour length, and stretching modulus of the curves are (A) 21.6 nm, 1.67 µm, and 1156 pN, (B) 36.0 nm, 0.96 µm, and 703 pN.

Lengths of S-Ena, as measured from TEM micrographs.

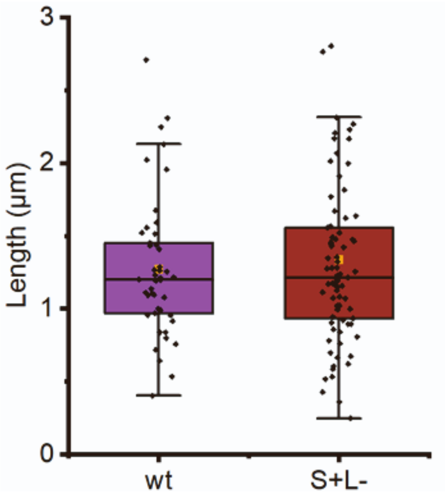

Figure S8. Measured lengths of the S-Ena pili in the wild-type (n = 46) and the S+L- (n = 82) knockout spores. There was no statistical difference between the samples (p = 0.60). The box plots show the 1st and 3rd quartiles (box limits), median values (lines), and 1.5·IQR (whiskers) for the pili lengths.

Performing multiple extension measurements

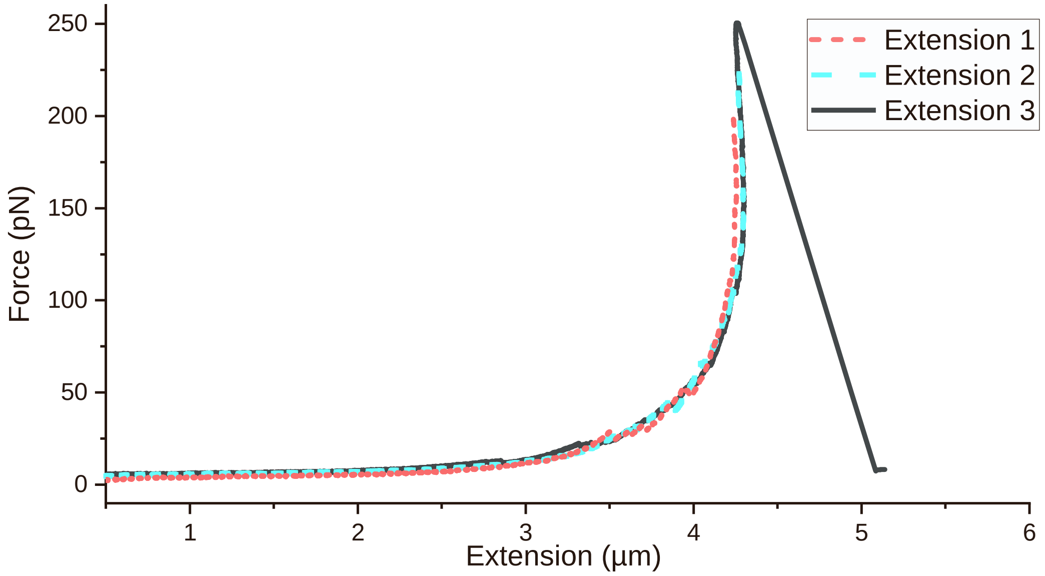

Figure S9. Three force extension measurements of S-Ena pili. For high forces the responses show a decrease in extension, this is related to the non-linear response of the trapped bead that occurs when applying too high forces. For the last measurement (black curve) the bead detaches from the pilus.

TEM micrographs of S+L- mutant spores

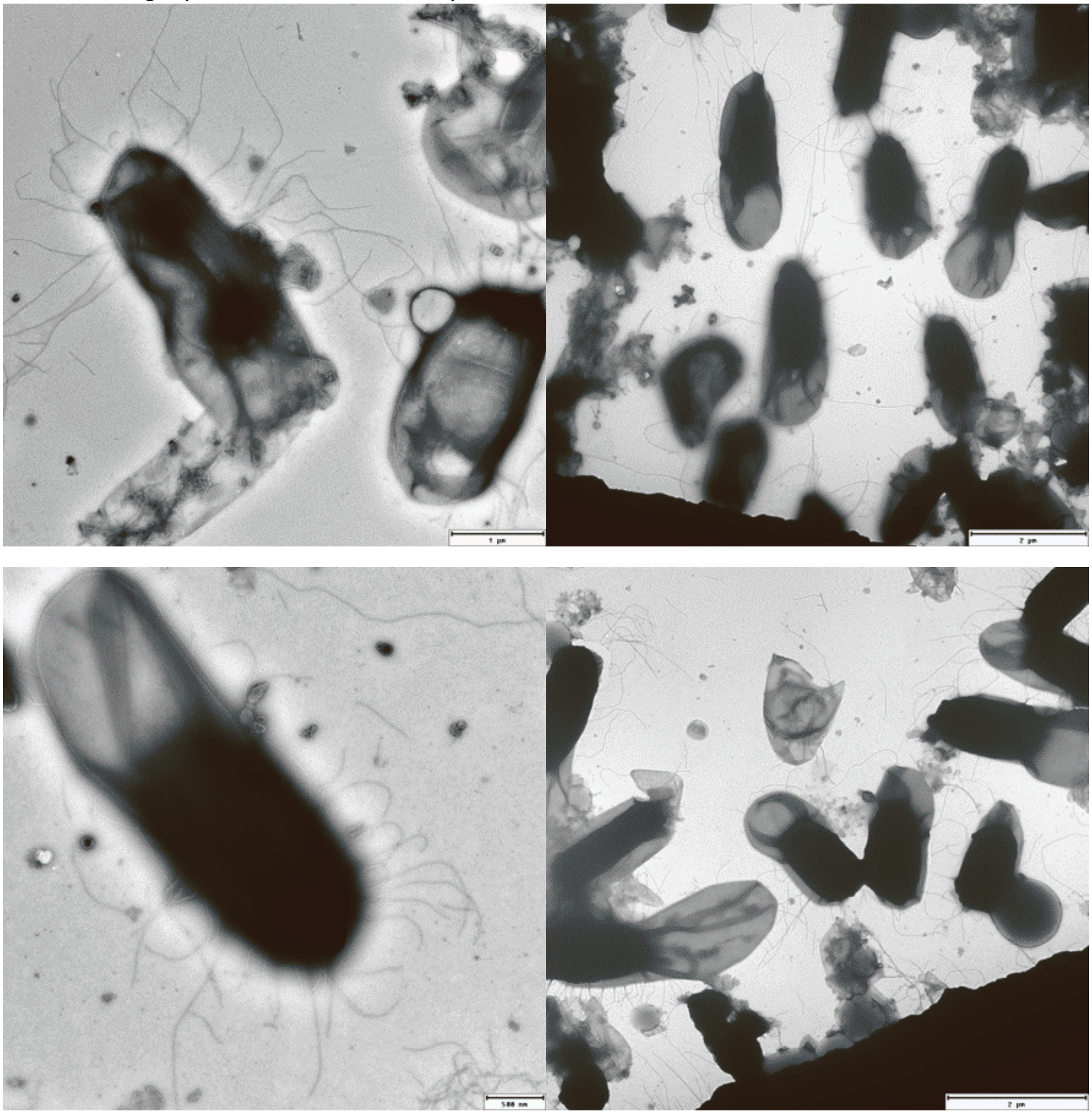

Figure S10. TEM micrographs of the S+L- *B. paranthracis* NVH 0075/95 mutant strain. The spores express S-type appendages but no L-type appendage.

Drag force simulation modeling

Drag force on spores is simulated using the Stokes drag force model, supplemented using the Goldman wall correction factor to model flows close to a surface. The drag force is thus modelled as

$$F = f\left(\frac{H}{R_e}\right) 6\pi\eta U R_e,$$

where η is the dynamic viscosity of the fluid, U is the undisturbed fluid velocity at the center of the spore, H is the distance between the surface and the spore center, and R_e is the effective hydrodynamic radius as calculated from our experimental data. The function $f\left(\frac{H}{R_e}\right)$ is the Goldman correction factor

$$f\left(\frac{H}{R_e}\right) = 0.700\left(\frac{H}{R_e}\right)^{-1.082} + 1.001$$

is fit from discrete data points as described by Wiklund *et al.* [2].

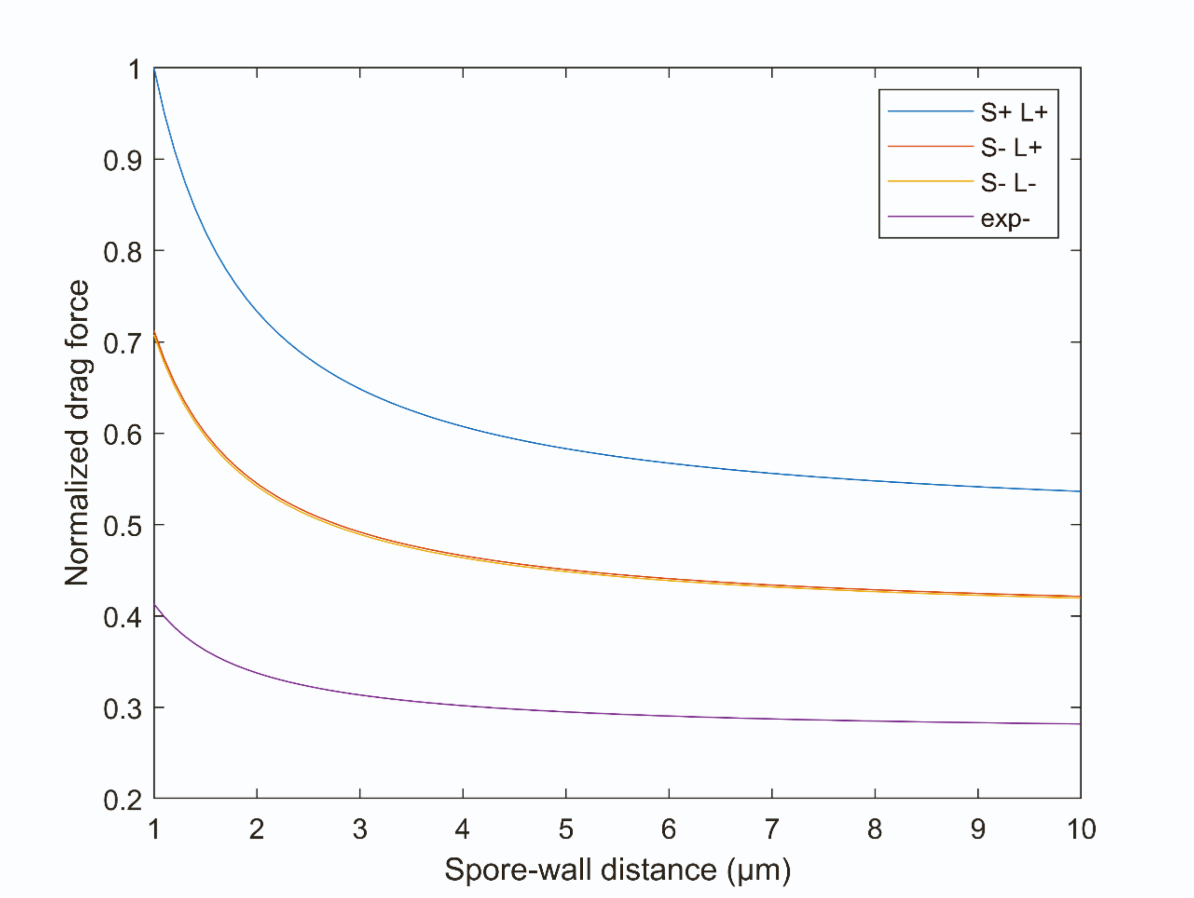

Figure S11. Normalized stokes shear force on the four studied *B. paranthracis* strains in a fluid flow, as a function of the spore-surface distance. We observe a significantly higher shear force on the S+ L+ strain compared to its Ena-, and exosporium deficient counterparts. Note the overlap in normalized drag force between the S- L+ and S- L- strain due to their similar effective hydrodynamic radii.

Supporting Movies

Movie S1. Strain lacking exosporium (exp-, lab. strain name 1521)

Movie S2. Strain lacking S and L Ena (S- L-, lab. strain name 1500)

Movie S3. Strain lacking S but expressing L (S- L+, lab. strain name 1489)

Movie S4. Strain expressing S and L Ena (S+ L+, lab. strain name 160)

Movie S5. Strain lacking L but expressing S (S+ L-, lab. strain name 1503)

Movie S6. Strain expressing extra long S and L Ena (S++ L+, lab. strain name 1487)

Movie S7. Strain expressing extra long S and L Ena (S++ L+, lab. strain name 1487)

References

1. Schindelin, J., Arganda-Carreras, I., Frise, E. et al. Fiji: an open-source platform for biological-image analysis. Nat Methods 9, 676–682 (2012).

2. Wiklund K., Zhang H., Stangner T., Singh B., Bullitt E., Andersson M. A drag force interpolation model for capsule-shaped cells in fluid flows near a surface. Microbiology; 164; 483-494 (2018).
